# How first-year medical students perceive changes in their approach to learning science

**DOI:** 10.3389/fmed.2026.1850900

**Published:** 2026-06-25

**Authors:** Moteeb Alhamidani

**Affiliations:** 1 King Abdullah International Medical Research Center, Riyadh, Saudi Arabia; 2 College of Science and Health Professions, King Saud bin Abdulaziz University for Health Sciences, Riyadh, Saudi Arabia; 3 Ministry of National Guard - Health Affairs, Riyadh, Saudi Arabia

**Keywords:** critical thinking, deep learning, first-year students, learning approaches, medical education, qualitative research, reflexive thematic analysis

## Abstract

**Background:**

Students enter medical education with established learning approaches shaped by years of prior schooling. How first-year medical students articulate perceived changes in these approaches during a course emphasizing critical thinking is not well understood. Understanding such perceptions offers insight into how students make sense of new academic demands, irrespective of whether behavioral change is measurable.

**Methods:**

This study used a qualitative descriptive design. Nine first-year medical students enrolled in a critical thinking–focused science course participated in semi-structured interviews lasting 30–50 min. Data were analyzed using reflexive thematic analysis applied at the semantic level, remaining close to participants’ own language and meanings.

**Results:**

Analysis generated four themes. First, most participants described a gradual shift from surface-level memorization toward attempts at deeper under standing. Second, the majority of participants reported developing more questioning stances toward course material. Third, several participants described efforts to map conceptual connections across lectures, finding these efforts meaningful but cognitively demanding. Fourth, most participants modified study habits, including increased use of self-testing, after discovering that prior methods were insufficient.

**Conclusion:**

These findings suggest that first-year medical students can articulate nuanced awareness of how their learning approaches may be shifting, even when the extent or permanence of such shifts remains uncertain. Perceived changes appear to reflect ongoing sense-making about what effective engagement with medical education requires.

## Introduction

In medical education, how students engage with learning material, not merely what they know, shapes their capacity for clinical reasoning and professional development (1–3). Students entering medical school carry learning approaches developed through years of prior education, including habitual strategies for memorizing, organizing, and applying knowledge (4, 5). Educational research has long distinguished between surface approaches, characterized by rote memorization and reproduction of content, and deep approaches, which involve seeking genuine understanding, interrogating evidence, and connecting new concepts to prior knowledge (6, 7). These approaches are not fixed personality traits; they are responsive to educational context, assessment demands, and the signals that teaching environments send about what kinds of engagement are valued (8, 9). Longitudinal research has confirmed this dynamism: medical students follow distinct trajectories of learning approach development across training, with a substantial proportion maintaining or increasing surface orientation over time (10).

The first year of medical school represents a particularly demanding educational transition. Students encounter teaching that rewards forms of engagement such as analysis, synthesis and clinical application, that may differ substantially from the strategies rewarded in prior contexts (11), and many enter with study skill deficits that become apparent only under the demands of the medical curriculum (12). Science courses in the first year often make this tension visible: they may require not only acquisition of factual knowledge but also its application and critique, which can prompt students to reconsider how they study (13). When courses explicitly identify critical thinking as a learning aim, this signals to students that certain forms of engagement are valued, though students may interpret those signals unevenly, and attempts to adjust their approaches may be gradual, partial, or inconsistent (8, 9). Recent reviews confirm that student-centered approaches such as problem-based learning can foster deeper engagement, though the conditions under which they do so remain incompletely understood (14–16). While much of the empirical evidence on learning approach transitions during clinical training comes from nursing education, comparable dynamics of adaptation and resistance have been observed in other health professions education contexts, and the mechanisms of transition appear broadly applicable (17–19).

Despite extensive research measuring learning approaches through validated instruments such as the Study Process Questionnaire and Approaches to Studying Inventory, less is known about how students themselves articulate awareness of changes in how they learn (20, 21). The relationship between students’ subjective perception of their own learning approaches and objectively measured changes in approach or academic performance is complex and not reducible to a simple correspondence (22). Students may describe noticing shifts in how they engage with material without those shifts manifesting as measurable score changes, and conversely, measured changes may occur without students being able to express them verbally. This gap between subjective and measured change is itself informative: it reveals how students interpret course demands and construct meaning from educational experiences. Research on metacognition in medical education has similarly shown that preclinical students often lack awareness of their own regulatory processes, suggesting that the capacity to give voice to learning approach perceptions is itself a developmentally significant competency (23).

Examining perceived change is therefore a legitimate and distinct object of inquiry. Students’ reflective accounts of their learning do not require validation through outcome measurement to constitute meaningful evidence about how educational contexts are experienced (24, 25). Studies in health professions education have found that students can describe their learning strategies with reasonable specificity, even when the depth or consistency of actual change varies (26, 27). What perceived change illuminates is students’ interpretive work, how they make sense of new academic contexts, what they notice about themselves, and what they believe is being asked of them. This interpretive process is part of the educational experience, not merely a secondary report on it.

The course context for this study was a required first-year science course at a health professions university. The course ran throughout the fall semester and combined weekly lectures with small-group, case-based discussions and problem-based activities requiring written analysis. Assessment included not only factual recall examinations but also written analytical assignments in which students were required to evaluate evidence and defend positions. Critical thinking was explicitly identified as a central learning aim, and students were made aware of this from the outset of the course. This design created conditions in which surface and deep forms of engagement were both possible and had differential consequences for performance, a context that the literature suggests is particularly likely to prompt awareness of learning approach (8, 28).

This study therefore aimed to explore how first-year medical students perceived changes in their approach to learning science in a course with explicit emphasis on critical thinking.

## Methods

### Research design

This study adopted a qualitative descriptive design (29, 30). Qualitative description aims to produce an account of participants’ experiences that remains close to the data and prioritizes their own language and meanings, without the degree of theoretical abstraction characteristic of phenomenology or grounded theory (31). This approach was chosen because the study aim was descriptive rather than theory-generative: to understand how students characterized perceived changes in their learning approaches, in their own terms. Reflexive thematic analysis, as developed by Braun and Clarke (32, 33), was used to identify patterns across participants’ accounts. Within this qualitative descriptive design, reflexive thematic analysis was applied at the semantic level: coding and theme construction remained close to participants’ own words and meanings, without moving toward latent or theoretical interpretation (32, 33). This use of RTA is consistent with a descriptive orientation, in which the researcher’s primary aim is to represent what participants said as faithfully as possible rather than to generate an interpretive account that goes beyond the data (29, 30). The researcher’s positionality was monitored throughout via a reflexive journal, not to introduce theoretical interpretation, but to guard against unconscious selective reading of the data through the lens of course outcomes. An independent qualitative researcher with expertise in health professions education research, external to the course and uninvolved in data collection, conducted an analytical audit of three transcripts selected to represent the breadth of thematic content in the dataset; one in which all four themes were prominent, one in which only two themes were evident, and one in which participants described limited or partial change. The reviewer read the transcripts and generated codes independently before comparing them with those of the primary researcher. Minor differences in code labelling were noted and resolved through discussion; no substantive disagreements in thematic structure arose, consistent with the semantic-level orientation of the analysis, which remained close to participants’ own language and afforded limited interpretive latitude.

### Participants and sampling

Nine first-year medical students participated in the study. All were enrolled in the course described above, the required first-year science course that explicitly identified critical thinking as a central learning aim, combined written analytical assessments with factual recall examinations, and thereby made the distinction between surface and deep engagement consequential for performance.

Participants were recruited through purposive sampling, applying the following inclusion criteria: current enrollment in the target course, completion of at least 70% of course activities, willingness to reflect on their learning processes, and no prior enrollment in a professional program (to ensure the first-year transition was genuinely novel for all participants). Exclusion criteria included: students who had previously failed and repeated the course, and students with <6 weeks of course exposure at the time of recruitment. Participants were recruited in the final 4 weeks of the semester, allowing sufficient course exposure for perceivable change while keeping the experience recent. Recruitment was conducted by the researcher through a written invitation distributed to all eligible students via the course learning management system. The invitation clearly stated that participation was entirely voluntary, that non-participation or withdrawal would have no bearing on course marks or grades. The sample of nine participants was appropriate for a qualitative descriptive study in which the goal was analytical depth rather than statistical generalizability.

### Data collection

Semi-structured interviews lasting 30–50 min were conducted individually with each participant in a private room by the author. The interview guide was developed through consultation with the literature on learning approaches and pilot-tested with two volunteer students not included in the study sample. The guide included opening questions about how students approached studying at the start of the semester; follow-up questions about whether and how their approach had changed; probes asking for specific behavioral examples; and reflective questions about what they attributed any changes to. Students were explicitly encouraged to describe what felt the same as well as what felt different, to avoid leading participants toward reporting only positive change. A summary of the interview guide domains is presented in Table 1. All interviews were audio-recorded and transcribed verbatim by a professional transcription service.

**Table 1:** Interview guide domains and representative questions.

Domain	Representative questions and probes
Opening: Baseline approach	“How did you typically go about studying at the start of this semester?”/“Can you walk me through what you did the last time you sat down to study for this course?”
Perceived change	“Has anything changed about how you approach studying since the start of the semester? If so, what has changed?”/“What has stayed the same?”
Behavioral examples	“Can you give me a specific example of a time when you noticed yourself studying differently?”/“What were you doing, and what prompted it?”
Attribution	“What do you think caused any changes you have noticed?”/“Were there particular aspects of the course that influenced how you studied?”
Reflection and continuity	“Do you think these changes will continue beyond this course?”/“Is there anything about how you studied before that you think worked better?”

### Data analysis

Transcripts were analyzed using Braun and Clarke (32, 33) reflexive thematic analysis. Analysis proceeded through six phases: familiarization (repeated reading of all nine transcripts and writing of initial impressions); initial coding (line-by-line open coding across all transcripts); theme construction (grouping codes into candidate themes, then reviewing and consolidating these into four themes); theme review (comparing themes against the coded data and the full transcripts to check that each theme was grounded in and representative of the data); theme definition (writing a clear narrative account of each theme); and write-up. Analytic decisions were documented in a reflexive journal throughout the process, the data underlying this study consist of interview transcripts from human participants collected under institutional review board approval. In accordance with participant consent agreements and institutional data governance requirements, raw transcripts cannot be made publicly available. De-identified excerpts supporting the reported findings may be made available from the corresponding author upon reasonable request, subject to ethical review and institutional approval.

## Results

Analysis of the nine interview transcripts generated four themes describing how participants perceived changes in their approach to learning science. The themes were: (1) moving from reproduction to understanding; (2) developing a questioning orientation; (3) building conceptual maps; and (4) restructuring study practices. These themes were not independent: most participants described related changes across multiple areas, and the themes represented distinct facets of what appeared to be a broader reorientation of engagement. Across all four themes, participants characterized perceived change as gradual and effortful, and noted that time pressure and workload could disrupt new approaches and prompt reversion to more familiar habits. The following sections present each theme with supporting evidence. All participant quotations are attributed by participant code (P1–P9).

### Theme 1: moving from reproduction to understanding

Most participants described a shift in what they aimed to do when studying, from reproducing content accurately to understanding why content mattered and how it connected to other knowledge. At the start of the semester, most described an approach centered on memorizing lecture slides and textbook passages.

*“I would just try to remember everything word-for-word from the slides. I thought that was doing well”* **(P3).**

Over time, participants described becoming more concerned with grasping mechanisms and principles. This shift was not presented as the abandonment of memorization but as a change in primary orientation. Memorization remained useful, but understanding was increasingly the goal that studying aimed to produce.

*“I started asking myself why this matters, not just what it is. I still need to remember things, but now I want to know what they mean”* **(P4).**

*“It’s like I went from trying to take a photograph to trying to understand the story behind the photograph. The facts are still there but they are attached to something”* **(P7).**

This shift was described as prompted by assessments that required explanation and application rather than recall alone. Several participants noted a specific moment when they realized their memorization-oriented approach was insufficient, often in a problem-based activity or written assignment, and described this as a turning point. However, most participants also noted that sustaining deeper engagement was effortful and that under conditions of high workload or time pressure, memorization felt more manageable.

*“When I’m stressed and the exam is close, I go back to just memorizing. I know it’s not the best way but it feels safer”* **(P8).**

### Theme 2: developing a questioning orientation

The majority of participants described developing a habit of questioning course material rather than accepting it as given. Before the course, most described treating assigned readings and lectures as authoritative sources to be absorbed. Over the course of the semester, they reported beginning to interrogate explanations, asking whether they were complete, whether alternatives existed, and whether evidence supported the claims being made.

*“Before I would just accept what the textbook said, but now I question whether that’s the only way to think about it. I look at other sources to see if they say the same thing”* **(P2).**

*“I catch myself now asking, ‘but why does it work that way?’ I did not used to do that. Before I thought asking why was a sign I did not understand”* **(P5).**

Participants attributed this shift to course activities that explicitly modeled questioning, small-group discussions in which the facilitator regularly asked ‘what is the evidence for that?’ and problem-based activities that required written evaluation of competing explanations. Several participants specifically described the facilitator’s questioning behavior as a model they began to internalize.

*“The way the facilitator never just accepted what we said made me realize that questioning wasn’t rude or a sign of failure. Now I do it to myself when I’m studying”* **(P1).**

Alongside the development of questioning, participants described increased awareness of the limits of their own knowledge, a kind of meta-awareness that was, for several participants, initially uncomfortable.

*“I realized I was questioning things I did not even have the background to evaluate properly. It felt overwhelming at first. But now it just means I know what I need to learn next”* **(P6).**

### Theme 3: building conceptual maps

Several participants described efforts to identify and represent connections between concepts that they had previously treated as separate. At the start of the semester, most described studying each lecture in isolation: reviewing, memorizing, and moving on.

*“I used to have a separate set of notes for each lecture. Everything was in its own box. I did not think about how they were connected”* **(P9).**

Over time, participants described deliberately trying to map relationships between concepts across lectures and units. This effort was described as cognitively demanding and not always successful, but as producing meaningful shifts in understanding when it worked.

*“I started making diagrams that tried to show how different things fitted together, how the mechanism we studied in week three actually explained what we saw in week six. When it clicked it felt completely different from just memorizing”* **(P3).**

*“I realized the concepts were all about the same underlying idea but presented in different ways. Once I saw that it became much easier to remember everything because it was one story, not fifteen separate things”* **(P7).**

Participants who described this theme most fully tended to identify a specific integrative activity, a problem that required applying knowledge from multiple lectures, or a moment in small-group discussion when a connection was made explicit by a peer, as the trigger for their recognition that conceptual mapping was possible and valuable.

*“There was a case in the small group where someone connected two things that I had thought were completely separate. That was the moment I thought, ‘Oh, that’s what I’m supposed to be doing’”* **(P4).**

A minority of participants described genuine difficulty finding time to pursue conceptual mapping under the demands of the course workload. For these participants, the approach was understood as aspirational rather than consistently practiced.

### Theme 4: restructuring study practices

Most participants described concrete changes to how they studied, including when, for how long, and with what methods. The most common change described was a shift from passive review to active recall and self-testing.

*“I stopped just reading over my notes and started doing practice questions to see if I actually understood. The difference in how much I retained was immediate”* **(P2).**

*“I started explaining concepts out loud, like I was teaching someone who did not know anything. If I could not explain it clearly, that told me I did not understand it yet”* **(P5).**

Other changes included revising earlier in the learning cycle rather than close to examinations, spending more time on actively difficult material rather than reviewing material that felt familiar, and seeking out explanatory resources, lectures from other institutions, textbooks with stronger conceptual framing, when the primary course materials did not produce understanding.

*“I now spend more time on the things I do not understand than the things I do. Before I used to review what I already knew because it felt good. Now I deliberately go to the hard parts”* **(P9).**

Participants described these practice changes as experimental rather than settled. Most framed their modified practices in tentative terms, noting that they were still uncertain which approaches were most effective for them. The majority of participants described reverting to familiar practices, particularly note review and passive re-reading, during high-pressure periods, and characterized this as a conflict between what they believed would be effective and what felt manageable.

*“I know self-testing works better but when I’m tired and I have five things to do, I just reread my notes because it’s easier. I’m still working out how to make the better habits stick”* **(P8).**

## Discussion

This study explored how first-year medical students perceived changes in their approach to learning science during a course explicitly emphasizing critical thinking. The theoretical framework underpinning this study draws on the surface-deep learning distinction (6, 7), which characterizes learning approaches as context-responsive orientations rather than fixed traits: deep approaches involve seeking genuine understanding, interrogating evidence, and connecting concepts, while surface approaches prioritize rote reproduction (6). “Perceived changes in learning approaches” are understood here as students’ subjective awareness of shifts in these orientations, how students themselves interpret and narrate changes in how they engage with learning material. This definition distinguishes perceived change from measured change: the former is a subjective, interpretive phenomenon that is informative in its own right, regardless of whether it corresponds to objectively quantifiable differences in approach scores or academic performance (22). The four themes generated, moving from reproduction to understanding, developing a questioning orientation, building conceptual maps, and restructuring study practices, collectively describe a perceived reorientation of learning engagement from surface-level reproduction toward more active, reflective, and integrative forms of involvement. This pattern is consistent with the proposition that educational context actively shapes students’ learning orientation rather than merely testing a pre-existing trait (8, 9, 34). The study extends existing accounts by demonstrating that first-year medical students can articulate learning approach awareness with considerable specificity, and can identify the course features and personal moments that they associate with perceived shifts, even when those shifts are incomplete, inconsistent, and vulnerable to disruption under workload pressure.

The finding that perceived change was described as gradual, effortful, and context-dependent aligns with evidence from health professions education that changes in learning orientation do not follow a linear developmental progression (17, 23, 35). Longitudinal analyses of learning approach patterns have shown that shifts toward deeper engagement are neither universal nor irreversible, and that academic performance is sensitive to these fluctuations (36). Participants in this study described what might be understood as oscillation, moving toward deeper engagement under conditions that supported it, and reverting to familiar surface strategies under conditions of stress or time scarcity. This oscillation has theoretical coherence: Biggs et al. (28) model of constructive alignment predicts that students’ approaches will respond to assessment and teaching design, and that where assessment rewards reproduction rather than understanding, surface approaches will be preserved or re-adopted. The present findings suggest that even when students perceive the value of deeper approaches and have experienced some success with them, the structural demands of the curriculum can undermine sustained engagement. This represents a systemic concern: perceived change in approach is not self-sustaining and may require ongoing architectural support from the curriculum.

The development of a questioning orientation, described by the majority of participants, is particularly noteworthy because it reflects not only a behavioral shift but a shift in epistemic stance. Participants described moving from treating course material as authoritative to interrogating it, noticing gaps, and seeking alternative explanations. This aligns with Perry (37) model of intellectual development, in which students move from dualistic acceptance of authority toward more relativistic, evidence-evaluating epistemologies, and with King and Kitchener’s (38) reflective judgment model. The present study offers situated qualitative evidence that this kind of epistemological movement can be perceived by first-year medical students within a single semester, though participants also described the movement as incomplete and dependent on specific contextual triggers. Notably, the development of questioning behavior appeared strongly contingent on facilitator modeling and on assessment practices that made interrogative engagement consequential for performance. Students who encountered facilitators who asked “what is the evidence for that?” described internalizing this as a personal habit; students whose assessments rewarded reproduction tended to revert to surface strategies under pressure. This suggests that the epistemological development described here is not simply a function of student disposition but is dependent on facilitation quality and assessment alignment. These factors are beyond the instructor’s individual influence and point to the need for institution-level investment in facilitator development and in assessment reform. If facilitator behavior is a primary lever for epistemological growth, then variation in facilitator competence and training becomes a significant equity concern: students taught by less questioning facilitators may miss the contextual triggers that others receive. This has direct implications for how facilitators are selected, trained, and supported: if students attribute their questioning behavior to having seen it modeled, then the quality of facilitation is not merely pedagogical but also epistemologically formative.

This study contributes to medical education research by demonstrating that students’ reflective accounts of perceived learning approach change constitute informative evidence about how the transition into medical education is experienced, not as a validated proxy for measured change, but as a distinct object of inquiry in its own right. How students interpret what is being asked of them, what they notice about themselves, and how they explain perceived shifts shapes how they engage going forward. This interpretive layer of educational experience has been relatively underexplored in medical education research, where learning approaches have predominantly been studied through quantitative instruments (20, 21). Qualitative descriptive designs, as methodological overviews have argued, are particularly well suited to capturing this kind of experiential evidence because they prioritize participants’ own language and meaning-making (31). Qualitative investigation of perceived change offers complementary insight that cannot be captured through questionnaire data alone: the texture of the shift, the moments that catalyzed it, and the conditions under which it falters.

These findings should be interpreted considering two contextual limitations. First, the study captures perceptions at a single point in time, near the end of one semester. How students’ learning approaches develop across the full arc of medical education, into clinical training, residency, and practice, remains beyond the scope of what can be inferred from these data. Second, the study was conducted in a single course with a specific design; the extent to which the findings generalize to courses with different pedagogical approaches is unclear. What the findings do suggest, with reasonable confidence, is that first-year medical students in a critically oriented course can and do engage in substantive self-reflection about their learning, and that this reflection, when elicited, is detailed, self-aware, and educationally meaningful.

## Implications

Several practical implications follow from these findings for medical educators and curriculum designers. First, making learning approach concepts explicit in early medical education may support students in articulating and reflecting on their own academic habits. Participants in this study often described having no prior language for the distinction between memorization-oriented and understanding-oriented engagement; providing this conceptual vocabulary early in the curriculum could enable more purposeful self-regulation.

Second, facilitator behavior appears to be a meaningful contextual trigger for students’ epistemological development. Training facilitators to model questioning, evaluate evidence openly, and make uncertainty visible may contribute to students’ development of a questioning orientation, an outcome with direct relevance to clinical reasoning. A systematic review of critical thinking pedagogical practices in medical education has catalogued a range of evidence-based strategies, including case discussion, error-based learning, and simulation, that facilitators can draw upon to cultivate this orientation (35).

Third, assessment design matters not only for what students learn but for how they approach learning. Assessments that reward application and analysis, alongside recall, appear to create conditions in which deeper engagement becomes a perceivable and achievable goal for students. Assessment reform toward this end supports not only better learning but a more generative relationship between students and course material. Empirical work on constructive alignment suggests that when students perceive clarity in intended learning outcomes and alignment between teaching and assessment, they are more likely to adopt deep approaches (39).

Fourth, the vulnerability of perceived change to workload pressure points to a structural problem: students may understand what more effective approaches look like but lack the time and cognitive bandwidth to implement them consistently. Curriculum design that actively manages cognitive load, particularly in the first year, may be as important for learning approach development as any specific pedagogical intervention. Reflective accounts from first-year students suggest that applying cognitive load principles to restructure study strategies can support more intentional learning practices (27).

Finally, students themselves can benefit from structured opportunities to reflect on their learning approaches, not as self-assessment exercises for grading, but as low-stakes, facilitated conversations about what they notice about how they study. Several participants described the interview itself as the first time they had articulated these perceptions explicitly, suggesting that the act of reflection was itself educational.

## Limitations

Several limitations bound the interpretation of these findings. The study’s small sample (*n* = 9) reflects the conventions of qualitative descriptive inquiry, in which sample size is justified by analytical depth rather than statistical representativeness. However, readers should note that the themes generated here reflect the perspectives of nine students in one institution and one course context, and should not be taken as representative of first-year medical students generally. The single institutional context further restricts transferability; the course’s explicit emphasis on critical thinking likely produced a context more conducive to learning approach reflection than courses with different orientations or assessment structures.

Additionally, this study captures perceptions at a single point in time, near the end of one semester. It offers no evidence about whether perceived changes persist, deepen, or erode over time. Longitudinal designs tracking the same students across subsequent semesters and into clinical training would be necessary to assess the durability of the learning approach shifts described here. Finally, the study elicits subjective perceptions of change; it does not measure whether these perceptions correspond to objectively different learning behaviors or outcomes.

## Conclusion

This study explored how first-year medical students perceived changes in their approach to learning science during a semester-long course emphasizing critical thinking. Four themes, moving from reproduction to understanding, developing a questioning orientation, building conceptual maps, and restructuring study practices, describe a perceived reorientation of learning engagement that participants characterized as gradual, effortful, and context-dependent.

The study’s distinctive contribution lies in demonstrating the specificity and self-awareness with which first-year medical students can articulate their learning approach perceptions, including the triggers that prompted perceived change and the conditions that disrupted it. This evidence matters for medical education not because it confirms that deep learning occurred, but because it reveals how students make sense of the demands placed on them, and how curriculum design, facilitation practice, and assessment can support or undermine that sense-making process.

Future research should examine whether perceived approach change at the beginning of medical education is associated with longer-term outcomes, including clinical reasoning development and professional identity formation, and what features of curriculum design most reliably sustain the reorientation that students in this study began to perceive. Meta-analytic evidence linking self-regulated learning strategies to academic and clinical outcomes in medical education (40) suggests that the study practices participants described restructuring may have downstream significance warranting longitudinal investigation.

## Data Availability

The raw data supporting the conclusions of this article will be made available by the authors, without undue reservation.
